# Bradford Infirmary

**Published:** 1892-12-17

**Authors:** 


### The Institutional Workshop.

### WITHIN THE HOSPITALS.

### BRADFORD INFIRMARY.

[By our Special Commissioner.]

Bradford is a city of hills, which reminds one in this respect of San Francisco, except that the hills are smaller, and that they are traversed by steam trams, instead of electrical cars. We much prefer the electrical cars, and believe that they might be usefully substituted for steam in Bradford, and elsewhere in this country.

The Bradford Infirmary has pursued its useful work for nearly seventy years. Since 1881 the in-patients have more than doubled, and there has been a steady and continuous increase in home and out-patients. The older portions of Bradford Infirmary leave something to be desired. They necessarily exhibit faults of construction which are surprising, but which can be readily remedied in the present day without excessive outlay. Each floor, for example, has only one bath, although it contains four good-sized wards, in addition to smaller ones. Such an arrangement causes much inconvenience, and renders certain useful ablutions somewhat difficult, if not impossible to enforce. The fittings, too, in the ward kitchens, are out of date, and might usefully be replaced.

It will scarcely be believed that the larger wards on each floor, with twenty-one beds, have no lavatory or "slop sink," in any proper meaning of that term. In the middle of each ward there are two doors which open into two cupboards, one of which contains a water closet and a tap for cold water, and the other two or three washing basins. Such a state of affairs is not defensible, as the hospital authorities admit; but the needful reconstruction has been delayed from a wish that any additional buildings should be so constructed as to avoid interference with the light and air of adjacent wards. So laudable a wish is worthy of commendation, but it ought not longer to prevent the carrying out of a plan of reconstruction which will supply each ward in the building with adequate lavatory, slop-sink, and bath accommodation of the best kind. We made a close examination of the buildings, and are satisfied that at relatively small expense, and without the least interference to the air or light of any other ward or building, it is possible to provide new lavatories, and to make them entirely adequate to the needs of each ward. We would suggest that some members of the Board of the Bradford Infirmary should visit the General Infirmary at Leeds, and see the new bath and lavatory arrangements there, because they combine in a minimum of space a maximum of efficiency, in addition to modern fittings and appliances, and an abundance of ventilation and fresh air.

The out-patients' department requires new flooring in the waiting-hall, but this, we understand, will shortly be supplied. We would suggest teak blocks, bedded in concrete. We would also suggest the addition of Rogers Field's automatic flushing tanks, one to be applied to each building, in order to secure that the whole of the drains are kept constantly flushed. From a casual inspection we made of one of the open man-holes, we came to the conclusion that at present the drains are very far from being free, and that they require prompt attention.

It would add much to the comfort of the patients if some one or more of the wealthier inhabitants of Bradford would take sufficient interest in the infirmary to make inquiries as to the latest and most suitable form of locker for the wards, and then to order enough to supply each bed with one throughout the infirmary. A suggestion of this kind made in Philadelphia produced all that was necessary within twenty-four hours, and we have confidence that the men of Bradford will be at least equally prompt. The ladies of Bradford might usefully take the nursing department in hand and use their influence to raise a fund for providing each nurse with a separate wash-hand stand, and also a supply of screens for those of the nurses' rooms which contain more than one bed, so that adequate privacy may be secured. There was also a marked absence of flowers at the the infirmary. We do not remember to have seen a hospital anywhere where there were so few flowers. We commend this fact to the attention of the Bradford ladies, in the hope that arrangements may be made to keep the wards bright with plants, ferns, and flowers, throughout each year.

In many hospitals it has now become the practice for some of the younger generation to organise a small society, and to arrange for some of their number to visit the hospital and give an entertainment in the wards. There is an American organ in the children's ward, and we gathered that anyone who is a competent performer, who would volunteer to play upon it periodically for the amusement of the children, would certainly not only be welcome, but useful and helpful too. We venture to mention this small matter because our experience proves that cheerfulness in the sick is the high road to recovery, and cheerfulness might, in our opinion, be helpfully promoted at Bradford to a greater extent than it is at present.

#### The New Buildings.

The new wards certainly leave little to desire. They are, of course, unusually large, but the superficial and cubic space is abundant. Cross ventilation is provided for, and the fittings, bath, and lavatory accommodation reflect great credit upon the authorities. We should have been glad to have seen some pictures, but these, no doubt, will be supplied, now that attention has been called to their absence. We are a little doubtful whether the plan of putting old and young children of both sexes promiscuously into one great ward containing thirty-one cots and beds is a good one. In the course of our inspection of the hospitals of England during the past six months we have been very much struck with the advantages attending a classification of the child patients as at Sheffield, Portsmouth, and elsewhere, which provides for the treatment of the boys in one ward and the girls in another. We would further point out that the supply of toys at Bradford is too limited, a fact which we commend to the attention of the ladies of the town. The health of sick children is greatly promoted by an adequate supply of toys, especially if they are kept outside the wards in a toyroom until after the doctors' visits. Such an arrangement secures to each child greater variety, and consequent happiness.

The condition of the wards was good. They were clean and well cared for, and the general appearance shows that the staff is really efficient. This is the more creditable to the nurses, seeing that in the larger wards the staff is undoubtedly too small. The big wards in the new building ought to have five nurses each, and to expect the work to be done by three is not only unreasonable, but unwise. There is no lift for patients in the new wards, and it would be very difficult to supply one, as the size of the staircase will not admit of the arrangement which has proved so successful at Sheffield. The operations theatre is well-lighted, and one of the best we have seen. We were glad to hear that the use of sponges a second time was discarded, and that they are not largely used at all.

### General Arrangements.

The general arrangements of the Bradford Infirmary are good. There is an evident desire to make steady progress in all directions, and that desire has been fruitful in results. The infirmary bakes its own bread, the Secretary possessing sufficient technical knowledge to enable him to select corn in the market, and ensure that such corn is of the best quality. We have never seen better bread than that baked at Bradford Infirmary. This is a most important matter, and we think many hospitals might usefully consider whether it would not be better for them bake their own bread rather than to have it supplied by contract. The sleeping accommodation and other arrangements for the comfort of the servants are good. Although situated in the basement, the cubicles are airy and, on the whole, well lighted, and we congratulate the authorities upon the attention and care they have displayed in this matter. There is an excellent lavatory, and bath-room for servants, a fact which we commend to other hospital authorities. All the two-bed cubicles ought to be supplied with a wash-hand basin for each bed, and sufficient screens to secure necessary privacy. Altogether we were impressed with the great interest taken in the work by the Secretary, and the marked intelligence and common sense which he displayed.

### The Nurses and Nursing.

At the Bradford Infirmary they have a system by which they train their own nurses. After a month's trial probationers are admitted for one complete year's training on the condition that they agree to continue in the service for at least two years longer, such engagement only to terminate by consent of the House Committee on written application, and on grounds which appear sufficient. In addition to board, lodging, washing, indoor uniform, and medical attendance, probationers receive £10 per annum, half of which is retained until the end of the engagement. Fourteen pounds is paid during the second year, and £18 during the third. If a probationer leaves without the sanction of the House Committee she forfeits £5, with the value of the uniform. At the end of three years' training such nurses as remain in the service receive a salary of £22 for the first year, which is increased to £25, and subsequently by increments of £1 to £30 per annum. Certificates are issued, after examination, at the end of three years. A system of lectures and training has been organised and is in force. In addition to the nurses trained for the Infirmary, a certain number of others are taken on for the Nurses' Institute. The Institute probationers remain for sixteen months at the hospital, but the Institute pays nothing to the hospital for the training. We are strongly of opinion that arrangements should be made to amalgamate the Nurses' Institute with the Infirmary, and that steps should be taken to erect a Nurses' Home of adequate proportions, wherein every member of the nursing staff, whether engaged in the hospital or on private cases, could be properly housed. At the present time the accommodation for the head or charge nurses is good. Each nurse has a separate bed-room with the use of a comfortable sitting-room. The probationers are provided with accommodation in a separate house. The sitting and dining-room arrangements are good, though the former is too small for so large a staff. Most of the probationers' bedrooms contain two beds, and it has been found necessary to utilise the attics of the old house in question, which are ill adapted for the continuous sleeping accommodation of young women who have to spend the greater part of each day in the vitiated atmosphere of a hospital. The infirmary authorities are quite alive to the importance of the points now urged, but as usual money is wanted to enable them to erect the necessary buildings. It would not be a bad idea for the Hospital Committee to raise the necessary capital to pay for a new Nurses' Home by bonds at four per cent., the interest of which should be guaranteed by the Hospital Committee, whilst the principal might be repaid by annual drawings out of the profits made by the private nursing staff. A similar course has been adopted in one or more hospitals with marked success. It would secure to the people of Bradford and the neighbourhood a supply of fully-trained, capable women, promote the comfort of themselves and their families when sick at home, and add much to the repute and efficiency of the nursing department of the Bradford Infirmary.

### A Word to Bradford's Citizens.

We hope the suggestions made in this article will be taken in good part, that they will be specially taken to heart by the people of Bradford, and so lead to the awakening of greater personal interest in the Infirmary, and all that concerns its best interests. In these days what our hospitals need is more personal service on the part of the residents in each town. Bradford is no exception to the general rule, and we believe that the defect in question is due to a want of thought, not to a want of heart. This emboldens us to believe that our suggestions, if acted upon, will prove fruitful in results, in the best interests of the poor of all classes, whilst well calculated to add to the benefits arising directly or indirectly from the work done within the walls of the Bradford Infirmary. Here, as at Sheffield, it is pleasant to notice in the Board-room a portrait of a late house surgeon who did good service to the Infirmary, and so received deserved and lasting recognition at the hands of the governors.

